# Genetic architecture of Environmental Sensitivity reflects multiple heritable components: a twin study with adolescents

**DOI:** 10.1038/s41380-020-0783-8

**Published:** 2020-06-03

**Authors:** Elham Assary, Helena M. S. Zavos, Eva Krapohl, Robert Keers, Michael Pluess

**Affiliations:** 1 Department of Biological and Experimental Psychology, School of Biological and Chemical Sciences, Queen Mary University of London, London, UK; 2 Department of Psychology, Institute of Psychiatry Psychology and Neuroscience, King’s College London, London, UK; 3 MRC Social Genetic and Developmental Psychiatry Research Centre, Institute of Psychiatry Psychology and Neuroscience, King’s College London, London, UK; 4 Centre for Economic Performance, London School of Economics, London, UK

**Keywords:** Genetics, Psychology

## Abstract

Humans differ substantially in how strongly they respond to similar experiences. Theory suggests that such individual differences in susceptibility to environmental influences have a genetic basis. The present study investigated the genetic architecture of Environmental Sensitivity (ES) by estimating its heritability, exploring the presence of multiple heritable components and its genetic overlap with common personality traits. ES was measured with the Highly Sensitive Child (HSC) questionnaire and heritability estimates were obtained using classic twin design methodology in a sample of 2868 adolescent twins. Results indicate that the heritability of sensitivity was 0.47, and that the genetic influences underlying sensitivity to negative experiences are relatively distinct from sensitivity to more positive aspects of the environment, supporting a multi-dimensional genetic model of ES. The correlation between sensitivity, neuroticism and extraversion was largely explained by shared genetic influences, with differences between these traits mainly attributed to unique environmental influences operating on each trait.

## Introduction

According to the recent evolutionary-inspired theories (i.e., differential susceptibility [1], biological sensitivity to context [2]), humans, like many other species [3], differ substantially in their sensitivity to contextual factors, with some more susceptible to environmental influences than others. Importantly, these theories suggest that heightened sensitivity predicts both the reactivity to adverse contexts as well as the propensity to benefit from supportive features of positive environments. In other words, sensitivity is proposed to influence the impact of environmental influences in a “for better and for worse” manner [4]. These prominent theories converge on the proposition that genetic factors play a significant role in individual differences in Environmental Sensitivity (ES) [1, 2, 5]. However, no studies to date have examined the heritability of ES in order to empirically test the proposed role of genetic factors. Therefore, the first aim of this study was to estimate the heritability of ES in a sample of 17-year old twins. The second aim was to examine the potential multi-dimensional genetic architecture of ES, informed by recent findings on the bifactor structure of the Highly Sensitive Child (HSC) scale [6]. Finally, we aimed to investigate the genetic overlap between ES and common personality traits.

The proposition that heightened sensitivity moderates the outcomes of environmental influences for better and for worse is supported by growing evidence showing that different markers of ES, such as child temperament [7], genetic variants [8] and physiological reactivity [9], moderate the impact of a wide range of environmental influences. Evidence in support of the genetic basis of ES is predominately drawn from gene by environment interaction (G × E) studies featuring both candidate gene [1, 10, 11] and genome-wide polygenic approaches [12].

Most current studies capture sensitivity indirectly, through statistical investigation of person–environment or gene–environment interactions. An alternative approach is to measure sensitivity more directly with the help of questionnaires that capture the typical behaviours and experiences of sensitive individuals. The Highly Sensitive Person (HSP) [5] and the HSC scales [6] are questionnaires that have been developed to measure such behavioural sensitivity, indexing sensitivity as a function of lowered threshold of reactivity to stimulation and greater depth and breadth of processing of sensory and emotional stimuli. Although the specific biological mechanisms underlying variations in ES as detected with the HSP and HSC scales are still unknown, variations in physiological stress reactivity [2], in dopaminergic and serotoninergic circuitry, and in the activity of brain regions involved in the depth and breadth of emotional and information processing such as the amygdala [1], appear to play a role [5].

Recent studies using these scales provide empirical evidence for the validity of these scales [13–15]. For example, children who scored higher on the HSC scale were found to benefit significantly more than less sensitive children from school-based resilience [16] and anti-bullying interventions [17]. Similar results emerged in the context of parenting quality, with more sensitive children being more affected by both negative and positive parenting practices regarding the development of externalizing problems and prosocial behaviour [18].

Importantly, while these sensitivity measures were initially conceptualised to reflect one general sensitivity factor, subsequent factor analyses identified three factors, each capturing different aspects of ES: Low Sensory Threshold (LST) reflecting variations in the threshold for reactivity to sensory stimuli; Ease of Excitation (EOE) manifested in being easily overwhelmed by contextual emotional psychological stimuli and Aesthetic Sensitivity (AES) characterised by greater attention to contextual details and aesthetic appreciation [15, 19, 20]. In consequent studies, a bifactor solution emerged as the best-fitting model for the data, with items loading on the three individual factors as well as on a general factor of sensitivity [6, 21]. These findings suggest that while the total score captures overall levels of sensitivity, the individual factors capture more specific aspects of sensitivity, with AES reflecting variations in sensitivity to more positive aspects of the environment, and EOE and LST reflecting variations in sensitivity to more negative contexts [6, 19, 20]. It is currently unclear how the genetic and environmental influences underlying ES give rise to a phenotype that reflects sensitivity to both negative and positive contexts. However, based on the detected bifactor structure of this scale, sensitivity to negative and positive influences may be a reflection of distinct genetic factors underlying two specific components, which, when present together in an individual, give rise to general sensitivity.

Higher sensitivity has been consistently associated with common personality traits, such as higher neuroticism and openness to experiences, and lower extraversion, with low-to-moderate effects sizes [6, 19, 20, 22]. Given that these personality traits are also known to be heritable [23, 24], the question that remains to be addressed is whether and to what degree the genetic architecture of ES overlaps with heritable components of common personality traits.

The current study applies quantitative behavioural genetics methodology in a large sample of 17-year old adolescent twins in order to examine (1) to what degree ES is heritable, (2) whether the heritability of sensitivity reflects a multi-dimensional structure, with genetic influences that are shared across the three components of ES, as well as influences that are distinct to each component and (3) the extent to which genetic and environmental influences on ES overlap with those on the Big Five personality traits.

## Methods

### Participants

The sample for the current study included a subset of adolescent twin pairs from the Twins Early Development Study (TEDS). TEDS is a large longitudinal epidemiological study of over 16,000 twin pairs born in England and Wales between 1994 and 1996. A detailed description of TEDS recruitment procedures and data has been provided elsewhere [25]. The data for the current study were obtained during one of the planned TEDS data collection waves. After excluding participants with severe medical disorders, history of perinatal complications, or unknown zygosity, the sample for the current study consisted of 2868 individuals (monozygotic (MZ) twins = 1011; same-sex dizygotic (DZ) twins = 901; opposite sex twins (OS) = 956) with HSC data. Big five personality data were available for 1156 of those individuals (MZ twins = 445; same-sex DZ = 354; OS twins = 357). The mean age of the participants upon returning the HSC and personality questionnaires was 17.06 (SD = 0.88) and 16.45 (SD = 0.26), respectively. Twins’ zygosity was determined via parental ratings of physical similarity, which is reported to be 95% accurate when compared with DNA analysis [26], as well as DNA testing in instances where zygosity could not be determined based on physical similarity.

### Measures

#### Environmental Sensitivity

ES was measured with the HSC scale [6], a 12-item self-report questionnaire devised specifically to measure sensitivity in children and adolescents. The scale measures participant’s endorsement of statements such as “When someone observes me, I get nervous. This makes me perform worse than normal”, and “I don’t like watching TV programs that have a lot of violence in them” on a Likert rating scale ranging from 1 = not at all to 7 = extremely. The scale comprises of three factors. The EOE factor is represented by items that related to becoming mentally overwhelmed by external stimuli (e.g. “I am annoyed when people try to get me to do too many things at once”), whereas LST is represented by items that relate to unpleasant sensory arousal to external stimuli (e.g. “Loud noises make me feel uncomfortable”). AES is reflected in items that relate to aesthetic awareness such as “I notice it when small things have changed in my environment”. Internal consistencies of the scales were comparable with that found in other studies [15, 19] with *α* = 0.81 for the main scale (HSC) and *α* = 0.64, 0.81 and 0.70 for AES, EOE and LST, respectively, (a copy of the questionnaire is available in Supplementary Information Table S1).

#### Five factor model rating form (FFMRF)

Personality was measured using an abbreviated five factor model questionnaire by Mullins-Sweatt et al. [27], containing short descriptors to define the personality traits of agreeableness, extraversion, neuroticism, openness to experience and conscientiousness. The 30 items of the questionnaire are organized in such a way that there are six items for each personality trait. Each item is rated on a Likert scale ranging from 1 to 5 (1 = extremely low and 5 = extremely high). For example, the anxiousness facet of neuroticism is rated from fearful/apprehensive to unconcerned/cool, and the ideas facet of openness is rated from strange/ creative to pragmatic/ rigid. The data on personality were administered online, along with other measures of that particular data collection wave. FFMRF is reported to be a reliable and brief measure of personality [28]. The internal reliability of the scale in our sample was in the acceptable range for each of the subscales of neuroticism (*α* = 0.71), extraversion (*α* = 0.72), openness (*α* = 0.63), agreeableness (*α* = 0.69), and conscientiousness (*α* = 0.77).

### Data analysis

To address the first aim of this study, a univariate ACE model was used to estimate the heritability of ES. An ACE model is constructed by using the inter-class correlations of MZ and DZ twins to estimate the contribution of genetic and environmental factors to observed phenotypic variations in a trait. An ADE model was also constructed and examined against the ACE model to determine the best-fitting model. In addition, sex differences in the heritability estimates were examined, using the main four sex-limitation models.

The ACE model partitions the phenotypic variance into additive genetic effects (A), shared/common environmental effects (C) and non-shared environmental effects (E). An ADE model, on the other hand, replaces the shared environmental effects (C) with non-additive genetic effects (D for dominance). Importantly, dominance genetic effects are only explored when there is no evidence for shared environmental effects (C). Shared environmental effects are the environmental influences that contribute to the similarity between twins, whereas non-shared environments are the environmental influences that make twins dissimilar such as individual-specific life events. The genetic correlation (rg) between MZ and DZ twin pairs is assumed to be 1 and 0.5, respectively, and the correlation between twins’ shared environments (rc) is assumed to be 1 for both MZ and DZ twin pairs. Higher phenotypic similarity within MZ twin pairs, in comparison with DZ twins, can therefore be attributed to MZ twins’ higher genetic similarity (A). Since C also contributes to the higher resemblance between MZ twin pairs, any variance not accounted for by A can be attributed to C (if the MZ correlation is more than twice the DZ correlation, non-additive genetic effects, such as dominance (D) are indicated). E is what makes twins different from one another and is estimated as the difference between the MZ twin correlations and 1. E also includes measurement error [29]. The sex-limitation models examined herein included: (1) qualitative sex differences, which examines differences in the sources of variation in males and females; (2) quantitative sex differences, which examines differences in the extent of influence of ACE parameters in males and females; (3) no sex differences but with phenotypic scalar, which includes a term to correct for phenotypic variance differences between males and females, but no differences in ACE influences between males and females and (4) homogeneity model, a reduced parameter model, where no sex differences exist in ACE estimates.

To address the second question, we constructed a multivariate common pathway ACE model (as well as a Cholesky decomposition ACE model [correlated factors solution] for comparison) to examine the genetic architecture of sensitivity as a function of its three components. To address the third question, we used a multivariate independent pathway ACE model to examine the extent to which the phenotypic correlation between ES and personality is due to common or specific genetic influences.

The multivariate models parse the variance/covariance of the phenotypes of interest into two sets of ACE effects: those that are due to shared ACE effects and those that are due to specific ACE effects for each phenotype. The common pathway model assumes that the shared ACE factors influence the variables of interest via a single psychometric/latent liability factor. The Cholesky decomposition-correlated factors model assumes that the phenotypic correlation between variables is due to correlating ACE influences, rather than via a latent factor. The structural equation modelling package of OpenMx [30] in R [31] was used to conduct all twin analyses.

## Results

### Descriptive statistics

Descriptive statistics including the sample size, mean scores and bivariate correlations for all measures are presented in Table 1. Females scored higher than males on sensitivity (total score of sensitivity: *F*_(1,1435)_ = 48.58, *p* < 0.001; EOE: *F*_(1,1435)_ = 25.56, *p* < 0.001; AES: *F*_(1,1435)_ = 14.64, *p* < 0.001; LST: *F*_(1,1435)_ = 54.42, *p* < 0.001) and personality measures of neuroticism (*F*_(1,561)_ = 0.16.93, *p* < 0.001), agreeableness (*F*_(1,558)_ = 11.40, *p* < 0.001) and conscientiousness (*F*_(1,560)_ = 7.09, *p* < 0.05). Mean differences were not statistically significant for openness (*F*_(1,560)_ = 0.06, *p* = 0.81) and extraversion (*F*_(1,560)_ = = 0.10, *p* = 0.32). Age was not significantly correlated with any of the traits, except for AES (*r* = 0.09, *p* < 0.001).

**Table 1:** Descriptive statistics of the study sample and all included variables.

	Sample	Mean (SD)		Bivariate correlations							
		Male	Female	HSC	EOE	AES	LST	N	O	C	E
ES	2868	45.16 (10.95)	49.23 (10.86)								
EOE	2868	17.77 (6.57)	19.55 (6.56)	0.88**							
AES	2868	20.30 (4.21)	21.11 (3.57)	0.58**	0.27**						
LST	2868	7.10 (3.70)	8.61 (4.00)	0.73**	0.52**	0.17**					
Neuroticism	1156	14.97 (4.20)	16.42 (4.17)	0.33**	0.39**	−0.02	0.24**				
Openness	1154	21.21 (3.90)	21.53 (3.57)	0.06	−0.02	0.19**	0.01	−0.02			
Conscientiousness	1150	21.81 (3.73)	22.68 (3.96)	−0.05	−0.13**	0.14**	−0.03	−0.16**	0.17**		
Extraversion	1154	21.53 (4.32)	21.45 (3.89)	−0.18**	−0.24**	0.13**	−0.21**	−0.38**	0.27**	0.29**	
Agreeableness	1152	21.18 (3.89)	22.31 (4.02)	0.01	−0.04	0.07	0.04	−0.19**	0.22**	0.35**	0.24**

Means and bivariate correlations represent the data from a sample of one randomly selected twin from each pair, to ensure data is not influenced by family relatedness. Bivariate correlations represent variables corrected for age and sex.

*ES* Environmental Sensitivity − total score, *EOE* ease of excitation, *AES* aesthetic sensitivity, *LST* low sensory threshold, *SD* standard deviation, *N* neuroticism, *O* openness, *C* conscientiousness, *E* extraversion.

*p < 0.01; **p < 0.001.

### Heritability of ES

Cross-twin correlations showed evidence of genetic influences on variability in all traits, with MZ twin correlations being larger than DZ twin correlations in both males and females (Table 2). Twin correlations differed across male and female pairs for all variables, but the univariate ACE sex-limitation model fitting results indicated no significant differences between sexes in ACE estimates for HSC and its three components. There was a slightly better fit of the phenotypic scalar model for LST and AES components (see Supplementary Information Tables S2 and S3 for univariate model fitting results). The heritability of ES was 0.47 (95% CI = [0.30, 0.53]), with no evidence of shared environmental influences. The remaining 0.53 (95% CI = [0.47, 0.59]) of the variation was due to non-shared environmental influences, which also includes measurement error. Comparing the ACE model fit with its sub models (AE, CE, E) indicated that the AE model was the most parsimonious, with no deterioration in fit compared with the full model (Δ−2ll = 0.0004, *p* = 0.98).

**Table 2:** Cross-twin correlations and ACE estimates for sensitivity and personality measures.

	Cross-twin correlations							ACE variance components (95% CI)		
	MZ	DZ	MZM	DZM	MZF	DZF	DZOS	A	C	E
ES	0.47 (0.41, 0.53)	0.24 (0.18, 0.30)	0.53 (0.42, 0.61)	0.24 (0.10, 0.37)	0.45 (0.36, 0.52)	0.26 (0.14, 0.36)	0.22 (0.14, 0.30)	0.47 (0.30, 0.53)	0.00 (0.00, 0.13)	0.53 (0.47, 0.59)
EOE	0.42 (0.32, 0.49)	0.22 (0.15, 0.27)	0.48 (0.36, 0.58)	0.35 (0.22, 0.46)	0.40 (0.30, 0.48)	0.27 (0.15, 0.38)	0.14 (0.06, 0.23)	0.42 (0.23, 0.48)	0.01 (0.00, 0.14)	0.58 (0.52, 0.65)
AES	0.39 (0.32, 0.46)	0.13 (0.09, 0.20)	0.42 (0.30, 0.51)	0.04 (−0.10, 0.17)	0.37 (0.27, 0.45)	0.19 (0.07, 0.29)	0.15 (0.06, 0.24)	0.36 (0.25, 0.42)	0.00 (0.00, 0.07)	0.64 (0.58, 0.71)
LST	0.41 (0.34, 0.48)	0.19 (0.13, 0.25)	0.48 (0.36, 0.58)	0.26 (0.12, 0.39)	0.39 (0.27, 0.47)	0.25 (0.13, 0.35)	0.13 (0.04, 0.22)	0.41 (0.27, 0.47)	0.00 (0.00, 0.00)	0.59 (0.53, 0.65)
NEU	0.33 (0.21, 0.43)	0.12 (0.00, 0.23)	0.30 (0.01, 0.50)	0.19 (−0.05, 0.40)	0.34 (0.21, 0.45)	0.13 (−0.09, 0.33)	0.08 (−0.09, 0.24)	0.31 (0.08, 0.41)	0.00 (0.00, 0.18)	0.69 (0.59, 0.79)
OPEN	0.40 (0.29, 0.50)	0.07 (−0.04, 0.19)	0.32 (0.09, 0.51)	0.04 (−0.20, 0.26)	0.43 (0.30, 0.54)	0.13 (−0.06, 0.31)	0.06 (−0.12, 0.23)	0.35 (0.24, 0.45)	0.00 (0.00, 0.00)	0.65 (0.55, 0.76)
CONS	0.32 (0.19, 0.43)	0.04 (−0.07, 0.15)	0.11 (−0.12, 0.33)	0.01 (−0.27, −0.28)	0.42 (0.27, 0.53)	0.03 (−0.14, 0.20)	0.06 (−0.12, 0.23)	0.26 (0.10, 0.37)	0.00 (0.00, 0.11)	0.74 (0.63, 0.85)
EXT	0.35 (0.24, 0.45)	0.24 (0.12, 0.35)	0.25 (0.02, 0.44)	0.20 (−0.08, 0.43)	0.39 (0.26, 0.50)	−0.08 (−0.31, 0.17)	0.39 (0.25, 0.51)	0.22 (0.00, 0.45)	0.13 (0.00, 0.35)	0.65 (0.54, 0.76)
AGR	0.27 (0.14, 0.38)	0.09 (−0.03, 0.20)	0.15 (−0.09, 0.36)	0.02 (−0.26, 0.29)	0.32 (0.17, 0.45)	0.09 (−0.07, 0.24)	0.12 (−0.08, 0.29)	0.25 (0.01, 0.35)	0.00 (0.00, 0.17)	0.75 (0.65, 0.87)

CIs not including 0 indicate significant estimates and non-overlapping CIs indicate significant difference between the estimates; twin correlations represent variables corrected for age and sex**.**

*ES* Environmental Sensitivity, *EOE* ease of excitation, *AES* aesthetic sensitivity, *LST* low sensory threshold, *NEU* neuroticism, *OPEN* openness, *CONS* conscientiousness, *EXT* extraversion, *AGREE* agreeableness, *MZ* monozygotic twins, *DZ* dizygotic twins, *MZM* monozygotic male twins, *MZF* monozygotic female twins, *DZM* dizygotic male twins, *DZF* dizygotic female twins, *DZOS* dizygotic opposite sex twins, *CI* 95% confidence interval, *A* additive genetic influences, *C* shared environmental influences, *E* non-shared environmental influences.

In order to examine dominant genetic effects, an ADE model was compared with the ACE model, but it was not found to be a better fit to the data (Δ−2ll = 0.0004, *p* = 0.98; parameter estimates: A = 0.48 95% CI [0.42, 0.56]; D = 0.00 95% CI [0.00, 0.27]; E = 0.52 95% CI [0.47, 0.58]), suggesting additive genetic influences sufficiently captured the heritability of ES.

### Genetic architecture of ES as a function of its three components

**Fig. 1:**
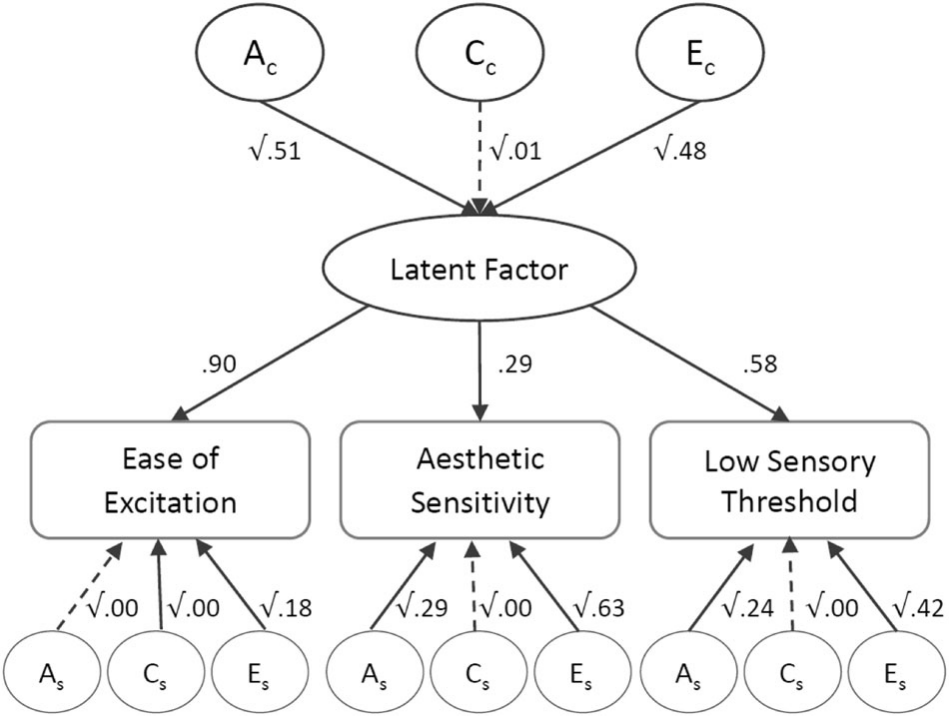
Common Pathway model, showing shared and specific genetic and environmental influences on the three components of sensitivity. Ac common additive genetic influences, Cc common shared environmental influences, Ec common non-shared environmental influences, As specific additive genetic influences, Cs specific shared environmental influences, Es specific non-shared environmental influences. The pathways from common ACE influences to the latent factor represent the standardized ACE estimates for the latent factor of sensitivity (A = 0.51, C = 0.01, E = 0.48). The pathways from the latent factor to the three components indicate the amount of variance explained in each component by the latent factor (ease of excitation = 90%, aesthetic sensitivity = 29%, low sensory threshold = 58%). The pathways from specific ACE influences to the components represent the standardized ACE estimates that are specific to each component. Dashed lines represent non-significant paths.

The common pathway model examined how much of the variance in the three components of sensitivity are due to common (Ac) versus specific genetic effects (As). The latent factor of sensitivity, as captured by EOE, AES and LST, was heritable (0.51, 95% CI = [0.29, 0.60]), with EOE loading most strongly on the latent factor (0.90, 95% CI = [0.83, 0.96]), followed by LST (0.58, 95% CI = [0.53, 0.63]) and AES (0.29, 95% CI = [0.25, 0.33]) (see Fig. 1). The proportion of variance explained by common and specific genetic and environmental influences on the three components is presented in Table 3. It was found that common genetic influences explained 0.42 (95% CI = [0.23, 0.48]) of the variance in EOE, 0.17 (95% CI = [0.10, 0.22]) of LST and 0.04 (95% CI = [0.02, 0.06]) of AES. Once common genetic influences were accounted for, there was no evidence of specific genetic influences on EOE, but 0.29 (95% CI = [0.20, 0.35]) and 0.24 (95% CI = [0.15, 0.29]) of the variation in AES and LST were explained by genetic influences specific to each component. This means that, whilst genetic influences on the heritability of EOE component were mainly explained by common genetic influences on the latent factor, 12% of the genetic influences on AES (calculated as 4/33) and 42% of the genetic influences on LST (calculated as 17/41) were explained by common genetic factors. The remaining heritability in AES and LST was due to genetic influences specific to each component (LST: 58% and AES: 88%).

**Table 3:** Results from common pathway model: shared and specific influences on the three components of ES.

	Common ACE influences			Specific ACE influences						
	Ac	Cc	Ec	As	Cs	Es				
Ease of excitation	0.42 (0.23, 0.48)	0.01 (0.00, 0.14)	0.39 (0.30, 0.50)	0.00 (0.00, 0.00)	0.00 (0.00, 0.00)	0.18 (0.09, 0.27)				
Aesthetic sensitivity	0.04 (0.02, 0.06)	0.00 (0.00, 0.01)	0.04 (0.03, 0.05)	0.29 (0.20, 0.35)	0.00 (0.00, 0.01)	0.63 (0.56, 0.69)				
Low sensory threshold	0.17 (0.10, 0.22)	0.00 (0.00, 0.06)	0.16 (0.13, 0.21)	0.24 (0.15, 0.29)	0.00 (0.00, 0.01)	0.42 (0.37, 0.48)				
Model fit summary for common pathway and Cholesky correlated factors solution										
	Models fit				Compared with saturated model			Compared with Cholesky		
	Parameters	−2ll	df	AIC	Δ−2ll	Δdf	*p*	Δ−2ll	Δdf	*p*
Fully saturated	135	49427.65	8469	32489.65						
Constrained	48	49504.15	8556	32392.15	76.50	87	0.78			
Cholesky correlated factors	26	49544.76	8578	32388.76	117.10	109	0.28			
Common pathway	23	49550.72	8582	32386.72	123.07	113	0.24	5.97	4	0.20

95% confidence intervals (CIs) are presented in brackets. CIs not including 0 indicate significant estimate.

*Ac* common A influences, *Cc* common C influences, *Ec c*ommon E influences, *As* specific A influences, *Cs* specific C influences, *Es* specific E influences, *fully saturated* model with maximum number of parameters describing the data, *constrained* the saturated model constrained to have the same mean and SD across twin and zygosity, *−2ll* minus twice the log likelihood, *df* degrees of freedom, *AIC* Akaike’s information criterion, *Δ−2ll* difference in −2ll value, *Δdf* difference in degrees of freedom, *p* *p* value.

Common non-shared environmental influences (Ec) explained 0.39 (95% CI = [0.30, 0.50]) of the variance in EOE, and 0.16 (95% CI = [0.13, 0.21]) and 0.04 (95% CI = [0.3, 0.5]) of the variance in LST and AES, respectively. Specific, non-shared environmental influences (Es) explained 0.18 (95% CI = [0.9, 0.27]), 0.63 (95% CI = [0.56, 0.69]) and 0.42 (95% CI = [0.37, 0.48]) in EOE, AES and LST, respectively.

A Cholesky decomposition [correlated factors solution] model was also fitted to the data to compare its fit with the common pathway model (See Supplementary Information Table S4). The common pathway model showed a better fit, as indicated by a lower AIC value, suggesting that a general factor of sensitivity captures the relationship between the three components better than three separate correlating factors (see Table 3).

Overall, results indicate that there are common genetic and environmental influences that underlie all three components of the sensitivity measure, contributing to a general factor of ES. At the same time, results indicate that there are also some specific genetic and environmental influences on the LST and AES components.

### Genetic overlap between ES and the Big Five personality traits

**Fig. 2:**
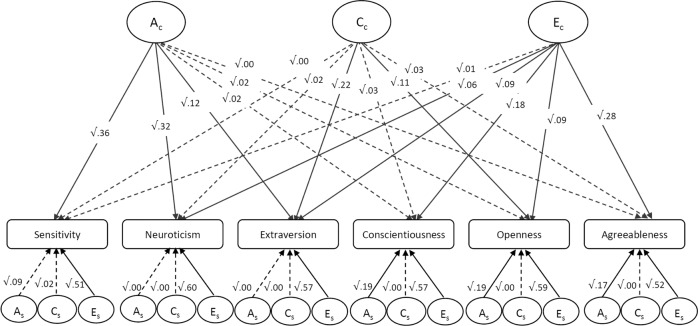
Independent Pathway model, showing shared and specific genetic and environmental influences on personality and sensitivity. Ac common additive genetic influences, Cc common shared environmental influences, Ec common non-shared environmental influences, As specific additive genetic influences, Cs specific shared environmental influences, Es specific non-shared environmental influences. The pathways from common ACE influences to sensitivity and personality represent the standardized variance components explained by common ACE influences in each trait. The pathways from specific ACE influences to sensitivity and personality traits represent the standardized ACE estimates that are specific to each component. Dashed lines represent non-significant paths.

The independent pathway model (Fig. 2) examined the proportion of variance of sensitivity and personality traits that were due to genetic effects that are common to all of them (Ac) versus those that are specific to each trait (As), and to those environmental influences that are common to all of them (Cc/Ec) versus those that are specific to each trait (Cs/Es). The results showed that common genetic influences (Ac) explain 0.36 (95% CI = [0.26, 0.51]) and specific genetic influences account for 0.09 (95% CI = [0.0, 0.27]) of the heritability of ES. Hence, of the total 0.45 heritability estimate for sensitivity in this model, 80% (calculated as 36/45) were due to genetic effects shared with personality traits, whereas the other 20% (calculated as 9/45) were due to genetic influences specific to sensitivity. Common genetic influences accounted for the entirety of the genetic influences on neuroticism (Ac = 0.32, 95% CI = [0.19, 0.42]) and extraversion (Ac = 0.12, 95% CI = [0.2, 0.27]), but did not make a significant contribution to the heritability of openness, conscientiousness or agreeableness (see Supplementary Information Table S5 for details). Therefore, the common genetic influences that explain individual differences in sensitivity are mainly shared with the personality traits of neuroticism and extraversion.

Common non-shared environmental influences (Ec) made a significant contribution to explaining the variance in all personality traits, but not in ES (Ec = 0.01, 95% CI = [0.00, 0.04]). Environmental influences that explained the variance in sensitivity were almost entirely (51/52 = 98%) due to non-shared environmental effects specific to this phenotype (Es = 0.51, 95% CI = [0.46, 0.59]). The small, non-significant effect of shared environmental influences on sensitivity (C) was due to effects specific to sensitivity (Cs = 0.02, 95% CI = [0.00, 0.14]).

Overall, these results suggest that the majority of the genetic influences that explain the heritability of sensitivity are shared with the personality traits of neuroticism and extraversion, while the environmental influences that explain individual differences in sensitivity are almost entirely specific to this phenotype. Of the total ACE influences on variations in sensitivity, 37% was explained by ACE effects shared with personality traits. The remaining 63% were due to ACE effects specific to sensitivity, indicating, although shared to a significant degree, a predominately distinct aetiology of sensitivity and personality traits.

## Discussion

The current study set out to examine three questions related to individual differences in ES. First, we investigated whether ES is a heritable trait. Second, we examined the genetic architecture of ES as a function of its three components and total score. Third, we investigated the extent to which the genetic and environmental factors that explain variability in the Big Five personality traits are shared with ES.

With regards to the first question we found that genetic influences accounted for 47% of the variation in sensitivity, while non-shared environmental influences and measurement error accounted for the remaining 53% of the variance. Our results support theories proposing that sensitivity is a heritable trait, whereby genetic variation explains nearly half of the observed individual differences in sensitivity.

In relation to the second question, the results of our analysis show that the genetic and environmental factors that explain variance in EOE and LST do not explain much of the variance in AES. These findings suggest that the genetic aetiology of ES is the function of three heritable components: one that is relevant to general sensitivity, as captured by the common genetic influences across the three components, another that is reflected in specific genetic influences that are involved in variations in the reactivity to adversity (i.e. LST and EOE components), and another that is relevant to processes involved in reactivity to positive experiences (i.e. AES component). Hence, our results support a multi-dimensional genetic model of sensitivity. An important implication of these finding is that the relative presence or absence of the specific genetic factors that contribute to the different heritable components of sensitivity may lead to different sensitivity types [32]. For example, some people may be more biased to react to adversity, due to having a higher proportion of the specific genetic factors that underlie the LST and EOE components. Others may show greater reactivity to positive aspects of the environment, due to carrying more of the specific genetic factors that contribute to the AES component. And people with LST/EOE-related genetic factors that also carry genetic factors related to AES will be more sensitive to both negative and positive environmental influences.

With regards to our final question, we found that sensitivity was moderately correlated with higher neuroticism and lower extraversion. The majority of the heritability of sensitivity was explained by genetic factors that also influence neuroticism, and to a lesser extent extraversion. A small proportion of the variance in sensitivity was explained by genetic factors that are specific to sensitivity. However, we found no evidence that environmental influences that are involved in the prediction of the Big Five personality traits are also relevant for variation in sensitivity. These findings suggest that the phenotypic similarities between ES, extraversion and neuroticism were largely due to their underlying shared genetic influences, whereas differences between these traits are predominately influenced by unique environmental factors.

The current study has several important strengths. These include the use of a twin design to provide a first estimate of heritability of ES in a large, representative sample of adolescent twins. Furthermore, this is the first study to examine shared heritability between ES and the Big Five personality traits. However, our findings have to be considered in light of the following limitations. First, it must be noted that the heritability estimates were based on an adolescent sample, and that these estimates may differ in younger or older samples given that heritability estimate often vary across the life course. Therefore, it cannot be assumed that the same estimates will necessarily apply to ES studies that feature infants and toddlers. Furthermore, it must be acknowledged that the obtained estimates are specific to the ES measure that we used. Although the HSC scale is a promising measure of ES, it may not fully capture systematic or heritable variance in ES and therefore other measures may indicate different heritability estimates. Future studies should seek to investigate and replicate the reported heritability estimates in different samples and at different ages (ideally featuring longitudinal designs), using additional and alternative measures of ES, as well as estimating heritability with molecular genome-wide methodology [33]. Second, all measures were based on self-report (although collected at different time points), which may have inflated observed correlation between them. Third, the subsample with personality measures was considerably smaller than the total sample, which may have prevented reliable detection of smaller effects. Fourth, we have to acknowledge that the limitations of twin design analyses [29, 34] also apply to this study, including the difficulty to detect effects of shared environments, which could have inflated our heritability estimates.

The results of the current study have several implications for future research. First, while our findings do not point to the specific genetic factors underlying variations in ES, they do provide assurance that ES, to the extent that is reflected in the HSC scale, is heritable. This should encourage its use as a proxy phenotype in molecular genetic research applying genome-wide and polygenic approaches. This is especially important given that much of the existing evidence suggesting that differences in ES are influenced by genetic factors is based on the widely criticised candidate gene methodology.

Second, we found evidence to suggest that the heritable components of ES is multi-dimensional and consists of three relatively distinct genetic influences: one that pertains to general sensitivity, one reflecting heightened sensitivity to negative and one to positive aspects of the context. Future studies of ES should investigate the existence and distribution of these hypothesized sensitivity components. Third, we found the majority of genetic influences involved in individual differences in ES are also involved in the personality traits of neuroticism and extraversion, encouraging future research on the shared genetic influences on these traits. Finally, we found that environmental factors also play a significant role in shaping ES, emphasizing the need for future research to examine the contribution of early environmental influences in the development of sensitivity.

In conclusion, the reported findings support the theoretical proposition that the phenotype of ES has a genetic basis, but that environmental factors play an equally important role. Furthermore, our findings suggest that ES, measured with the HSC scale, reflects a multi-dimensional genetic structure made up of three heritable components. Finally, the genetic overlap between ES, neuroticism and extraversion indicates that similar genetic influences are involved in these different phenotypes.

## Supplementary information

Supplemental Information
