# Supplementary material for: Genetic architecture of Environmental Sensitivity reflects multiple heritable components: a twin study with adolescents
Source: Mol Psychiatry. 2020 Jun 3;26(9):4896–904. doi: 10.1038/s41380-020-0783-8 (PMC8589650; doi:10.1038/s41380-020-0783-8)
Supplement: Supplementary file 1 — Supplemental Information [file 41380_2020_783_MOESM1_ESM.docx]

**Supplementary Information**

**Genetic Architecture of Environmental Sensitivity Reflects Multiple Heritable Components: a Twin Study with Adolescents**

Elham Assary^1^, PhD; Helena M.S. Zavos^2^, PhD; Eva Krapohl^3^, PhD; Robert Keers^1^, PhD; Michael Pluess^1, 4*^, PhD

^1^ Department of Biological and Experimental Psychology, School of Biological and Chemical Sciences, Queen Mary University of London, UK.

^2^ Department of Psychology, Institute of Psychiatry Psychology and Neuroscience, King’s College London, UK.

^3^ MRC Social Genetic and Developmental Psychiatry Research Centre, Institute of Psychiatry Psychology and Neuroscience, King’s College London, UK.

^4^ Centre for Economic Performance, London School of Economics, UK.

* All correspondences to be addressed to Michael Pluess at:

Department of Biological and Experimental Psychology

School of Biological and Chemical Sciences, Queen Mary University of London
G.E. Fogg Building, Office 2.01

Email: [m.pluess@qmul.ac.uk](mailto:m.pluess@qmul.ac.uk)

**Table S1.**

Highly Sensitive Child (HSC) Questionnaire

INSTRUCTIONS: Answer each question according to the way you personally feel, using the following scale:

**1** 2 3 **4** 5 6 **7**

**Not at All Moderately Extremely**

1. I notice when small things have changed in my environment

2. Loud noises make me feel uncomfortable

3. I love nice smells

4. I get nervous when I have to do a lot in little time

5. Some music can make me really happy

6. I am annoyed when people try to get me to do too many things at once

7. I don’t like watching TV programs that have a lot of violence in them

8. I find it unpleasant to have a lot going on at once

9. I don’t like it when things change in my life

10. I love nice tastes

11. I don’t like loud noises

12. When someone observes me, I get nervous. This makes me perform worse than normal

| **Table S2.**  Univariate ACE Models Fit Summary for Environmental Sensitivity and its three components | | | | | | | | | | |
| --- | --- | --- | --- | --- | --- | --- | --- | --- | --- | --- |
|  | **Model** | **Model fit** | | | | | | | | |
|  |  | Compared to Fully Saturated Model | | | | | | Compared to Quantitative ACE Model | | |
|  |  | **-2ll** | **df** | **AIC** | **Δ -2ll** | **Δ df** | **p** | **Δ -2ll** | **Δ df** | **p** |
| Sensitivity | Fully Saturated | 21736.95 | 2843 | 16050.95 | - | - | - | - | - | - |
|  | Constrained | 21752.89 | 2859 | 16034.89 | 15.95 | 16 | 0.46 | - | - | - |
|  | Qualitative ACE (rg=Free) | 21752.98 | 2859 | 16034.98 | 16.03 | 16 | 0.45 | 0 | 1 | 0.99 |
|  | Qualitative ACE (rc=Free) | 21752.98 | 2859 | 16034.98 | 16.03 | 16 | 0.45 | 0 | 1 | 1 |
|  | Quantitative ACE | 21752.98 | 2860 | 16032.98 | 16.03 | 17 | 0.52 | - | - | - |
|  | Scalar ACE | 21754.8 | 2862 | 16030.8 | 17.85 | 19 | 0.53 | 1.82 | 2 | 0.4 |
|  | **ACE -no Scalar** | **21756.23** | **2864** | **16028.23** | **19.28** | **21** | **0.57** | **3.25** | **4** | **0.52** |
| Ease of Excitation | Fully Saturated | 18871.71 | 2843 | 13185.71 | - | - | - | - | - | - |
|  | Constrained | 18889.26 | 2859 | 13171.26 | 17.55 | 16 | 0.35 | - | - | - |
|  | Qualitative ACE (rg=Free) | 18889.33 | 2859 | 13171.33 | 17.62 | 16 | 0.35 | 1.05 | 1 | 0.31 |
|  | Qualitative ACE (rc=Free) | 18889.26 | 2859 | 13171.26 | 17.55 | 16 | 0.35 | 1.12 | 1 | 0.29 |
|  | Quantitative ACE | 18890.38 | 2860 | 13170.38 | 18.67 | 17 | 0.35 | - | - | - |
|  | Scalar ACE | 18898.34 | 2862 | 13174.34 | 26.63 | 19 | 0.11 | 7.96 | 2 | 0.02 |
|  | **ACE -no Scalar** | **18901.46** | **2864** | **13173.46** | **29.75** | **21** | **0.1** | **11.08** | **4** | **0.03** |
| Aesthetic Sensitivity | Fully Saturated | 15810.66 | 2843 | 10124.66 | - | - | - | - | - | - |
|  | Constrained | 15832.27 | 2859 | 10114.27 | 21.6 | 16 | 0.16 | - | - | - |
|  | Qualitative ACE (rg=Free) | 15837.51 | 2859 | 10119.51 | 26.85 | 16 | 0.04 | 0.15 | 1 | 0.7 |
|  | Qualitative ACE (rc=Free) | 15837.66 | 2859 | 10119.66 | 27 | 16 | 0.04 | 0 | 1 | 1 |
|  | Quantitative ACE | 15837.66 | 2860 | 10117.66 | 27 | 17 | 0.06 | - | - | - |
|  | **Scalar ACE** | **15837.89** | **2862** | **10113.89** | **27.23** | **19** | **0.1** | **0.23** | **2** | **0.89** |
|  | ACE- no scalar | 15864.5 | 2864 | 10136.5 | 53.84 | 21 | <.001 | 26.84 | 4 | <.001 |
| Low Sensory Threshold | Fully Saturated | 15871.03 | 2843 | 10185.03 | - | - | - | - | - | - |
|  | Constrained | 15878.88 | 2859 | 10160.88 | 7.86 | 16 | 0.95 | - | - | - |
|  | Qualitative ACE (rg=Free) | 15878.89 | 2859 | 10160.89 | 7.86 | 16 | 0.95 | 0 | 1 | 1 |
|  | Qualitative ACE (rc=Free) | 15878.88 | 2859 | 10160.88 | 7.85 | 16 | 0.95 | 0 | 1 | 0.95 |
|  | Quantitative ACE | 15878.89 | 2860 | 10158.89 | 7.86 | 17 | 0.97 | - | - | - |
|  | **Scalar ACE** | **15884.53** | **2862** | **10160.53** | **13.51** | **19** | **0.81** | **5.65** | **2** | **0.06** |
|  | ACE- no scalar | 15899.83 | 2864 | 10171.83 | 28.81 | 21 | 0.12 | 20.94 | 4 | <.001 |
| *Note.* Fully Saturated model=model with maximum number of parameters describing the data; Constrained = sub-model of the fully saturated model, testing the assumptions of twin design, with means and variances equated across twins and zygosity; Qualitative ACE (rg=Free) and Qualitative ACE (rc=Free) = models that allow differences in source of variation in males and females, where either rC or rG is free to be estimated for opposite sex twin pairs and can vary below the values assigned to same-sex dizygotic pairs; Quantitative ACE =model that allows differences in the extent of influence of ACE parameters in males and females, with rC and rG in opposite sex twins being fixed to 1 and .5 respectively, estimating the ACE parameters from same sex twin pairs only; Scalar ACE = model with no sex differences in ACE parameters but scalar term on males; ACE - no Scalar= univariate ACE model with no difference between males and females;−2ll= minus twice the log likelihood; df= degrees of freedom; AIC= Akaike’s information criterion; Δ AIC= difference in AIC value; Δ -2ll =difference in -2LL value; Δ df= difference in degrees of freedom; p= p-value; The best fitting models are marked as bold, selected based on the principle of parsimony and lowest AIC and -2ll value. A difference in AIC between two models of 2 or less, provides equivalent support for both models, in which case the most parsimonious model (i.e. with lowest number of parameters) was chosen, a difference of 3 indicates that the lower AIC model has considerably more support, and a difference of more than 10, indicates that the lower AIC model is a substantially better fit compared to the higher AIC model. | | | | | | | | | | |

| **Table S3.**  Univariate ACE Models Fit Summary for Personality Traits | | | | | | | | | | | |
| --- | --- | --- | --- | --- | --- | --- | --- | --- | --- | --- | --- |
|  | **Model** | **Model fit** | | | | | | | | | |
|  |  |  |  |  |  |  |  |  |  |  |  |
|  |  | Compared to Full Saturated Model | | | | | | Compared to Quantitative ACE Model | | | |
|  |  | **-2ll** | **df** | **AIC** | **Δ -2ll** | **Δ df** | **p** |  | **Δ -2ll** | **Δ df** | **p** |
| Neuroticism | Fully Saturated | 6559.76 | 1131 | 4297.76 | - | - | - |  | - | - | - |
|  | Constrained | 6582.64 | 1147 | 4288.64 | 22.89 | 16 | 0.12 |  | - | - | - |
|  | Qualitative ACE (rg=Free) | 6582.76 | 1147 | 4288.76 | 23.01 | 16 | 0.11 |  | 0.04 | 1 | 0.84 |
|  | Qualitative ACE (rc=Free) | 6582.80 | 1147 | 4288.80 | 23.05 | 16 | 0.11 |  | 0.00 | 1 | 1.00 |
|  | Quantitative ACE | 6582.80 | 1148 | 4286.80 | 23.05 | 17 | 0.15 |  | - | - | - |
|  | Scalar ACE | 6583.78 | 1150 | 4283.78 | 24.02 | 19 | 0.20 |  | 0.98 | 2 | 0.62 |
|  | **ACE -no Scalar** | **6583.84** | **1152** | **4279.84** | **24.09** | **21** | **0.29** |  | **1.03** | **4** | **0.90** |
| Openness | Fully Saturated | 6207.96 | 1129 | 3949.96 | - | - | - |  | - | - | - |
|  | Constrained | 6224.07 | 1145 | 3934.07 | 16.11 | 16 | 0.45 |  | - | - | - |
|  | Qualitative ACE (rg=Free) | 6225.66 | 1145 | 3935.66 | 17.70 | 16 | 0.34 |  | 0.91 | 1 | 0.34 |
|  | Qualitative ACE (rc=Free) | 6226.57 | 1145 | 3936.57 | 18.61 | 16 | 0.29 |  | 0.00 | 1 | 1.00 |
|  | Quantitative ACE | 6226.57 | 1146 | 3934.57 | 18.61 | 17 | 0.35 |  | - | - | - |
|  | Scalar ACE | 6228.96 | 1148 | 3932.96 | 21.00 | 19 | 0.34 |  | 2.39 | 2 | 0.30 |
|  | **ACE -no Scalar** | **6233.20** | **1150** | **3933.20** | **25.24** | **21** | **0.24** |  | **6.63** | **4** | **0.16** |
| Conscientiousness | Fully Saturated | 6270.45 | 1125 | 4020.45 | - | - | - |  | - | - | - |
|  | Constrained | 6289.14 | 1141 | 4007.14 | 18.68 | 16 | 0.29 |  | - | - | - |
|  | Qualitative ACE (rg=Free) | 6292.78 | 1141 | 4010.78 | 22.33 | 16 | 0.13 |  | 0.08 | 1 | 0.78 |
|  | Qualitative ACE (rc=Free) | 6292.85 | 1141 | 4010.85 | 22.40 | 16 | 0.13 |  | 0.00 | 1 | 1.00 |
|  | Quantitative ACE | 6292.85 | 1142 | 4008.85 | 22.40 | 17 | 0.17 |  | - | - | - |
|  | Scalar ACE | 6301.70 | 1144 | 4013.70 | 31.25 | 19 | 0.04 |  | 8.85 | 2 | 0.01 |
|  | **ACE -no Scalar** | **6298.06** | **1146** | **4006.06** | **27.61** | **21** | **0.15** |  | **5.20** | **4** | **0.27** |
| Extraversion | Fully Saturated | 6389.55 | 1129 | 4131.55 | - | - | - |  | - | - | - |
|  | Constrained | 6406.31 | 1145 | 4116.31 | 16.76 | 16 | 0.40 |  | - | - | - |
|  | Qualitative ACE (rg=Free) | 6410.77 | 1145 | 4120.77 | 21.22 | 16 | 0.17 |  | 7.22 | 1 | 1.00 |
|  | Qualitative ACE (rc=Free) | 6417.99 | 1145 | 4127.99 | 28.44 | 16 | 0.03 |  | 0.00 | 1 | 1.00 |
|  | Quantitative ACE | 6417.99 | 1146 | 4125.99 | 28.44 | 17 | 0.04 |  | - | - | - |
|  | Scalar ACE | 6418.57 | 1148 | 4122.57 | 29.03 | 19 | 0.07 |  | 0.58 | 2 | 0.75 |
|  | **ACE -no Scalar** | **6421.47** | **1150** | **4121.47** | **31.93** | **21** | **0.06** |  | **3.49** | **4** | **0.48** |
| Agreeableness | Fully Saturated | 6208.85 | 1127 | 3954.85 | - | - | - |  | - | - | - |
|  | Constrained | 6239.35 | 1143 | 3953.35 | 30.49 | 16 | 0.02 |  | - | - | - |
|  | Qualitative ACE (rg=Free) | 6240.17 | 1143 | 3954.17 | 31.32 | 16 | 0.01 |  | 0.00 | 1 | 1.00 |
|  | Qualitative ACE (rc=Free) | 6240.17 | 1143 | 3954.17 | 31.32 | 16 | 0.01 |  | 0.00 | 1 | 1.00 |
|  | Quantitative ACE | 6240.17 | 1144 | 3952.17 | 31.32 | 17 | 0.02 |  | - | - | - |
|  | Scalar ACE | 6241.82 | 1146 | 3949.82 | 32.96 | 19 | 0.02 |  | 1.65 | 2 | 0.44 |
|  | **ACE -no Scalar** | **6243.96** | **1148** | **3947.96** | **35.11** | **21** | **0.03** |  | **3.79** | **4** | **0.43** |
| *Note*. Fully Saturated model=model with maximum number of parameters describing the data; Constrained = Sub-model of the fully saturated model, testing the assumptions of twin design, with means and variances equated across twins and zygosity; Qualitative ACE (rg=Free) and Qualitative ACE (rc=Free) = model that allows differences in source of variation in males and females, where either rC or rG is free to be estimated for DZOS twin pairs and can vary below the values assigned to same-sex DZ pairs; Quantitative ACE =model that allows differences in the extent of influence of ACE parameters in males and females, with rC and rG in DZOS being fixed to 1 and .5 respectively, estimating the parameters from same sex twin pairs only; Scalar ACE = model with no sex differences in ACE parameters but scalar term on males; ACE - no Scalar= univariate ACE model with no difference between males and females; df= degrees of freedom AIC= Akaike’s information criterion; Δ -2ll =difference in -2LL value; Δ df= difference in degrees of freedom; p= p-value; The best fitting models are marked as bold, selected based on the principle of parsimony and lowest AIC and -2ll value. A difference in AIC between two models of 2 or less, provides equivalent support for both models, in which case the most parsimonious model (i.e. with lowest number of parameters should be chosen), a difference of 3 indicates that the lower AIC model has considerably more support, and a difference of more than 10, indicates that the lower AIC model is a substantially better fit compared to the higher AIC model | | | | | | | | | | | |

**Table S4.**

Correlated Factors and Common Pathway Models fit summary

| **Model Results: Correlated Factors** | | | | | | | | | | | | | |
| --- | --- | --- | --- | --- | --- | --- | --- | --- | --- | --- | --- | --- | --- |
|  | A correlation | C correlation | E correlation | | Phenotypic correlation | | Phenotypic correlation due to A | | Phenotypic correlation due to C | | | Phenotypic correlation due to E | |
| EOE - AES | 0.45 (.23,.67) | 1 | 0.14 (.07,.22) | | 0.27 (.23,.30) | | 0.17 (.07,.24) | | 0.01 (-.03,.08) | | | 0.09 (.04,.14) | |
| EOE - LST | 0.62 (.43,.78) | 1 | 0.45 (.39,.51) | | 0.52 (.49,.55) | | 0.25 (.12,.32) | | 0.01 (-.03,.08) | | | 0.26 (.22,.32) | |
| AES - LST | 0.34 (.14,.56) | 1 | 0.06 (-.01,.13) | | 0.17 (.13,.21) | | 0.13 (.04,.19) | | 0.01 (-.03,.08) | | | 0.04 (-.01,.08) | |
| **Models’ Fit Summary: Common Pathway and Correlated Factors** | | | | | | | | | | | | | |
|  | | Models Fit | | | | | | | | Compared to Saturated Model | | | |
|  | | estimated parameters | | -2ll | | df | | AIC | | Δ -2ll | Δ df | | p |
| Full Saturated | | 135 | | 49427.65 | | 8469 | | 32489.65 | |  |  | |  |
| Constrained | | 48 | | 49504.15 | | 8556 | | 32392.15 | | 76.50 | 87 | | 0.78 |
| Correlated Factors Model | | 26 | | 49544.76 | | 8578 | | 32388.76 | | 117.10 | 109 | | 0.28 |
| Common Pathway Model | | 23 | | 49550.72 | | 8582 | | 32386.72 | | 123.07 | 113 | | 0.24 |
|  | |  | |  | |  | |  | | Compared to Correlated Factors Model | | | |
|  | | | | | | | | | | 5.97 | 4 | | 0.2 |
| Common Pathway Model | | | | | | | | | |  |  |  |  |
| *Note.* EOE=Ease of Excitation; AES= Aesthetic Sensitivity; LST=Low Sensory Threshold; Full Saturated= model with maximum number of parameters describing the data; A = additive genetic influences; C = shared environmental influences; E = non-shared environmental influences; Constrained= The saturated model constrained to have the same mean and SD across twin and zygosity; −2ll= minus twice the log likelihood; df= degrees of freedom AIC= Akaike’s information criterion; Δ -2ll =difference in -2LL value; Δ df= difference in degrees of freedom; p= p-value | | | | | | | | | | | | | |

| **Table S5.**  Independent Pathways ACE Model fit results | | | | | | | | |
| --- | --- | --- | --- | --- | --- | --- | --- | --- |
| Model | Model fit | | | |  | Fit compared to the saturated model | | |
|  | estimated parameters | -2ll | df | AIC |  | Δ -2ll | Δ df | p |
| Fully Saturated | 450 | 52397.45 | 8184 | 36029.45 |  |  |  |  |
| Constrained | 165 | 52717.82 | 8469 | 35779.82 |  | 320.37 | 285 | 0.07 |
| Independent Pathways ACE model | 48 | 52908.85 | 8586 | 35736.85 |  | 511.4 | 402 | <.001 |
| Fully Saturated= model with maximum number of parameters describing the data; Constrained = the saturated model constrained to have the same mean and SD across twin and zygosity; −2ll= minus twice the log likelihood; df= degrees of freedom AIC= Akaike’s information criterion; Δ -2ll =difference in -2LL value; Δ df= difference in degrees of freedom; p= p-value | | | | | | | | |
